# *Listeria monocytogenes* Infection Affects a Subset of Ly49-Expressing NK Cells in the Rat

**DOI:** 10.1371/journal.pone.0015579

**Published:** 2010-12-15

**Authors:** Hamid Shegarfi, Christian Naper, Bent Rolstad, Marit Inngjerdingen

**Affiliations:** 1 Department of Anatomy, Institute of Basic Medical Sciences, University of Oslo, Oslo, Norway; 2 Institute of Immunology, Oslo University Hospital, Rikshospitalet, Oslo, Norway; Centre de Recherche Public de la Santé (CRP-Santé), Luxembourg

## Abstract

NK cells are protective against certain bacterial and viral infections, and their production of IFN-γ is important for the early innate immune defence against *L. monocytogenes*. We have previously shown that depletion of NK cells in rats leads to increased bacterial burden upon *L. monocytogenes* infection, and that a subset of NK cells encompassing the majority of Ly49 receptors (Ly49s3^+^ NK cells) contributed to this effect. In this study, we have further investigated how the Ly49s3^+^ NK cell subset is affected by *L. monocytogenes* infection. We observed an increased percentage of Ly49s3^+^ NK cells in the spleen and a reduction in the bone marrow within the first 48 hrs of *L. monocytogenes* infection. Concomitantly, we observed increased expression levels of the inflammatory chemokine receptors CCR5 and CXCR3 by Ly49s3^+^ bone marrow NK cells, as compared to Ly49s3^−^ NK cells, suggesting involvement of Ly49s3^+^ NK cells in the early phase of infection. However, NK cell production of IFN-γ was independent of Ly49 receptor expression. Furthermore, we observed increased expression levels of MHC class I molecules on both macrophages and NK cells during the first 48 hrs of infection, paralleled by a reduction in the surface expression of Ly49s3 on NK cells. In conclusion, *L. monocytogenes* infection modulates the tissue distribution of Ly49s3^+^ NK cells, and induces increased MHC class I expression and hence reduced surface expression of Ly49 receptors on NK cells. These changes indicate that *L. monocytogenes* infection may have multiple effects on NK cells *in vivo*, and suggests the involvement of Ly49-expressing NK cells in the immune responses towards *L. monocytogenes*.

## Introduction

NK cells are important contributors to the early immune defence against infections, and a protective factor against certain transformed cells [1]. Upon encountering target cells, NK cells mount a swift cytotoxic response, and release pro-inflammatory cytokines and chemokines. This response is controlled by numerous NK cell receptors, with both activating and inhibitory functions. The C-type lectin-like receptor families Ly49, CD94/NKG2, and NKR-P1 play important roles for rodent NK cells. These receptors are all encoded by genes within the Natural Killer Cell complex (NKC), a gene complex found in all mammalian species so far investigated. Ly49 receptors are functional homologues of human Killer Immunoglobulin-like Receptors (KIRs), and both receptor families are highly polymorphic. Both Ly49 and CD94/NKG2 receptor families recognize major histocompatibility complex (MHC) class I molecules, while NKR-P1 receptors bind C-type lectin-related (Clr) molecules [2]. All three families include inhibitory and activating receptors. In the rat, Ly49 receptors show specificity for both classical (RT1-A) and non-classical (RT1-CE) MHC class I molecules [3]–[7]. The surface expression levels of Ly49 receptors are under influence of MHC class I ligands expressed on neighbouring cells (interaction *in trans*) and NK cells themselves (interaction *in cis*) [8]. This implies that Ly49 receptors are highly expressed in rodent strains lacking their appropriate MHC class I ligands, and down-modulated when ligands are present [9], [10]. MHC class I molecules are constitutively expressed by almost all cell types, but their expression level is reduced in response to a number of viral and bacterial infections. This may render them sensitive to NK mediated lysis, by a mechanism where NK cells are relieved from inhibition through their inhibitory receptors [11]. However, the expression levels of MHC class I molecules may also increase in response to some viral infections and inflammatory stimuli such as type 1 and 2 interferons (IFN) and tumor necrosis factor-alpha (TNF-α) [12]–[14].

*Listeria monocytogenes* is a Gram-positive facultative intracellular bacterium that may cause sepsis and meningitis in immune-compromised individuals and severe foetal infections in pregnant women. It primarily infects epithelial cells and macrophages, and has a unique intracellular life cycle that allows spreading from cell to cell without being exposed to the extracellular environment [15]. Upon *L. monocytogenes* infection, infected macrophages secrete a number of inflammatory cytokines, such as TNF-α, interleukin-12 (IL-12), and chemokines that lead to the activation and recruitment of macrophages, neutrophils, NK cells, and γδ T cells that control the infection until adaptive T-cell responses eventually clear the bacteria and provide sterile immunity [16]–[21]. IL-12 and TNF-α may act in synergy to promote production of IFN-γ by innate lymphocytes, such as NK cells [22], [23]. However, in the course of an infection, IFN-γ production by NK cells may also require additional signals provided by direct cell-to-cell contacts with infected cells [23]–[25]. IFN-γ promotes the generation of fully activated, listericidal macrophages. The importance of IFN-γ is evident in mice lacking the IFN-γ receptor, which are highly susceptible to *L. monocytogenes* [26]. Activated macrophages display increased levels of major MHC class II molecules and produce listericidal free radicals, such as nitric oxide [15].

In the rat, two major subsets of NK cells can be distinguished by their complementary expression of the Ly49s3 receptor or the inhibitory NKR-P1B receptor [27], [28]. Expression of most Ly49 receptors is confined to the Ly49s3^+^ NK cell subset. We have previously demonstrated a protective role of NK cells in the early phase of *L. monocytogenes* infection in rats, possibly implicating the Ly49s3^+^ subset [29]. We have here investigated how *L. monocytogenes* infection affects the Ly49s3^+^ NK cell subset. We found that infection with *L. monocytogenes* led to an accumulation of Ly49s3^+^ NK cells in the spleen, with a simultaneous decrease in the bone marrow with accompanying changes in chemokine receptor expression. While IFN-γ production by NK cells appeared to be Ly49-independent, *L. monocytogenes* infection led to reduced surface expression of the Ly49s3 receptor, concomitant with increased expression of their MHC class I ligands by both macrophages and NK cells during the first 48 hrs of infection. We suggest that Ly49 receptors may be involved in the NK cell responses to *L. monocytogenes* infection, possibly influencing NK mediated killing of infected cells.

## Results

### Accumulation of Ly49s3^+^ NK cells in the spleen of *L. monocytogenes*-infected rats

**Figure 1:**
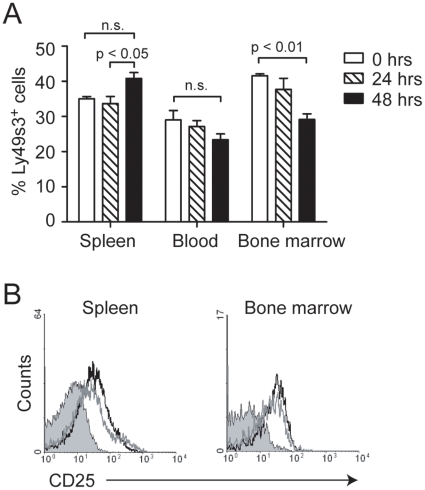
The percentage of Ly49s3^+^ NK cells increases in the spleen and decreases in the bone marrow following *L. monocytogenes* infection. A) Mononuclear leukocytes were harvested from spleen, blood, and bone marrow from uninfected and infected animals 24 and 48 hrs p.i. Cells were stained with antibodies to NKR-P1A, CD3, and Ly49s3. NK cells were gated as NKR-P1A^+^CD3^−^ cells, and the percentage of Ly49s3^+^ NK cells was analysed by flow cytometry. The data represent the mean ± SEM from three independent experiments, with 2 animals at each time point. Data were analyzed with the one-way ANOVA with Tukey's multiple comparisons test. B) The activation status of NK cells from uninfected (filled histogram) or infected animals 24 (grey line) and 48 hrs p.i. (black line) was tested by flow cytometry. An antibody towards the activation marker CD25 was employed.

We have previously reported a slightly increased percentage of Ly49s3^+^ NK cells, a subset encompassing the majority of Ly49 receptors, in the spleen of rats infected with *L. monocytogenes* [29]. Here, we have further investigated how the Ly49s3^+^ NK cell population is influenced during the first 48 hrs of infection by *L. monocytogenes*. We first assessed the percentages of Ly49s3^+^ NK cells in the spleen, blood, and bone marrow before and after i.v. infection with *L. monocytogenes*. While the percentage of Ly49s3^+^ NK cells was increased in the spleen 48 hrs after infection, we observed a simultaneous reduction in the bone marrow and possibly also in blood (Fig. 1A). *L. monocytogenes* infection also appeared to activate NK cells, as an increased surface expression of the activation marker CD25 (the high affinity IL-2 receptor alpha chain) was observed (Fig. 1B). No difference in CD25 expression was observed between Ly49s3^+^ and Ly49s3^−^ NK cells (data not shown).

### NK cells in the spleen and bone marrow modulate chemokine receptor expression after *L. monocytogenes* infection

**Figure 2:**
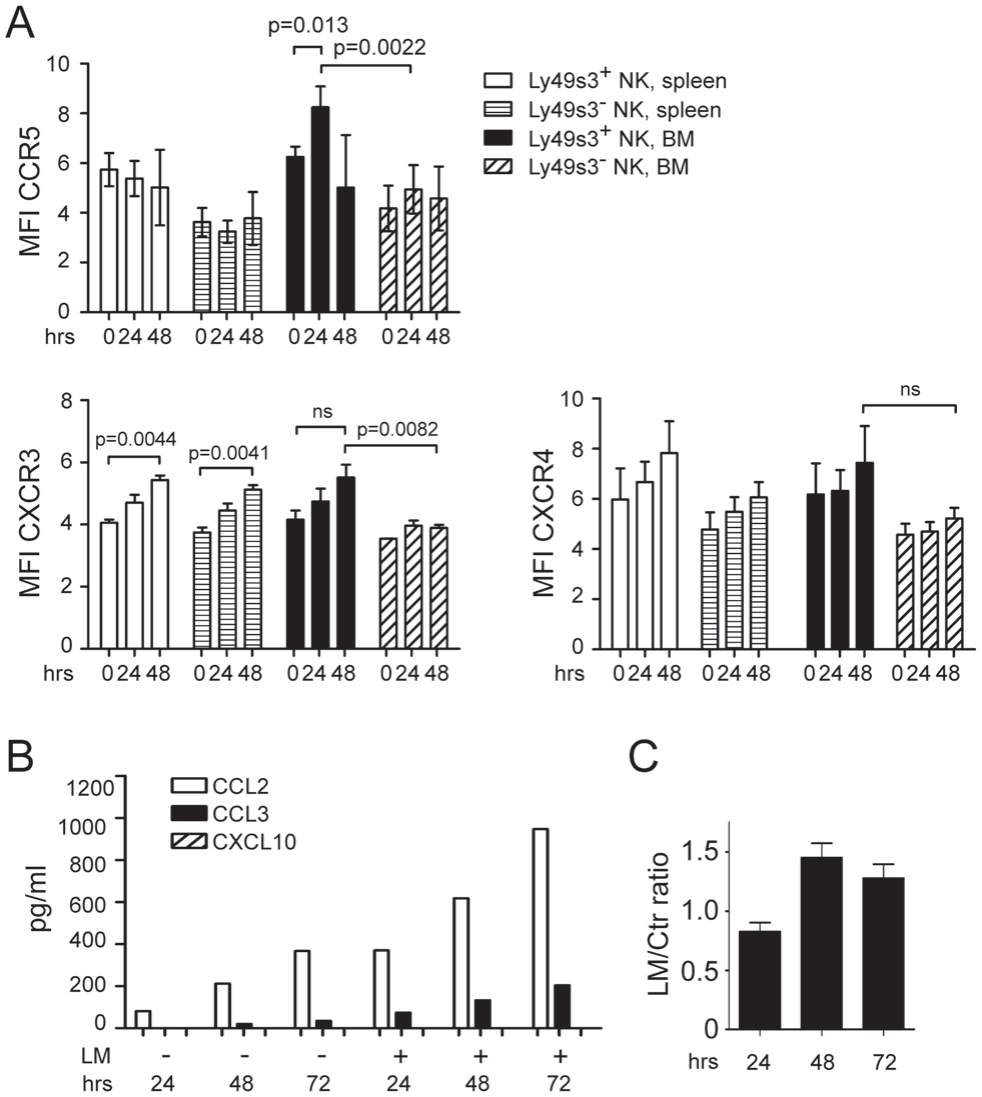
*L. monocytogenes*nfection modulates chemokine receptor expression *in vivo* and increases chemokine secretion by macrophages *in vitro.* A) Spleen and bone marrow cells were harvested from uninfected or infected animals, and stained for flow cytometry. NK cells were defined as NKR-P1A^+^CD3^−^, further gated as either Ly49s3^+^ or Ly49s3^−^ NK cells. The median fluorescence intensities of CCR5, CXCR3, or CXCR4 by Ly49s3^+^ or Ly49s3^−^ NK cells at 0, 24, and 48 hrs p.i. are presented. The data represents three individual experiments with two animals at each time point, ± SEM. B) The release of chemokines by uninfected or *L. monocytogenes* infected R2 macrophages was analyzed in supernatants harvested at 24–72 hrs of culture with a multiplex cytokine assay. C) The ability of supernatants from infected R2 macrophages to induce NK cell chemotaxis was analysed in a Transwell chemotaxis assay. Supernatants from uninfected macrophages were used as negative controls. The data are presented as the ratio of migrated cells in response to infected supernatants versus uninfected supernatants. Mean ± SEM from three independent experiments performed in duplicates. *p* = 0.0075 (one way ANOVA with Tukey's multiple comparisons test).

The observed changes in the distribution of Ly49s3^+^ NK cells upon *L. monocytogenes* infection prompted us to test whether the expression of chemokine receptors on NK cells was altered upon infection. Rats were infected with *L. monocytogenes* and NK cells from the spleen and bone marrow were analyzed 24 or 48 hrs p.i. A small, but reproducible, increase in the expression of the inflammatory chemokine receptor CXCR3 was observed on both splenic Ly49s3^+^ and Ly49s3^−^ NK cells 48 hrs p.i. (Fig. 2A). In the bone marrow, CXCR3 expression was increased amongst Ly49s3^+^ NK cells as compared to Ly49s3^−^ NK cells (Fig. 2A). Furthermore, we found that Ly49s3^+^ NK cells expressed marginally higher levels of CCR5 than Ly49s3^−^ NK cells in the bone marrow 24 hrs p.i. (Fig. 2A). Expression of the chemokine receptors CXCR4 and CCR2 remained unchanged on NK cells both in the spleen and bone marrow (Fig. 2A, and data not shown).

We next examined whether *L. monocytogenes* infected macrophages produced chemokines that could attract NK cells. To test this, we infected the rat macrophage cell line R2 with *L. monocytogenes*. Utilizing a multiplex cytokine assay, we detected higher levels of CCL2 (a CCR2 ligand) and CCL3 (a CCR1/CCR5 ligand) in the supernatants of infected macrophages versus uninfected macrophages (Fig. 2B). No secretion of CXCL10 (a CXCR3 ligand) was detected (Fig. 2B). Importantly, supernatants harvested from infected macrophages were able to induce chemotaxis of freshly isolated splenic NK cells, with maximum responses to supernatants harvested 48 and 72 hrs p.i. (Fig. 2C). This suggests that NK cells may also respond to chemokines secreted by infected macrophages *in vivo*.

### Splenic NK cells display an activated phenotype, and produce IFN-γ in a cell contact dependent manner after *L. monocytogenes* infection

**Figure 3:**
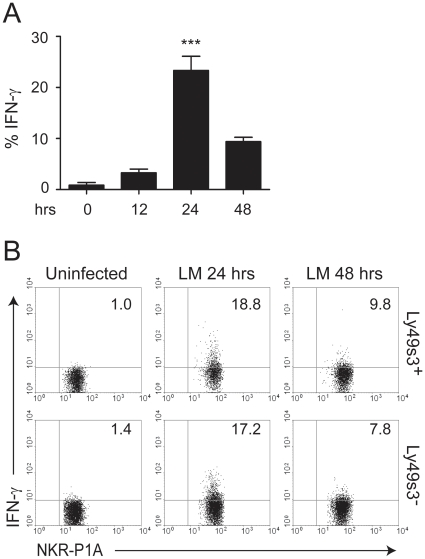
Production of IFN-γ in response to *L. monocytogenes* infection is Ly49-independent. Splenocytes from rats either uninfected or infected with *L. monocytogenes* for 24–48 hrs were harvested and the production of IFN-γ by (A) bulk NK cells or (B) Ly49s3^+^ or Ly49s3^−^ NK cells was determined by flow cytometry. The data in A) represents the mean ± SEM of two independent experiments, with two animals in each group (n = 4). Data were analyzed with the one-way ANOVA with Tukey's multiple comparisons test, ***: *p*<0.001.

IFN-γ production by NK cells is thought to be a key factor controlling *L. monocytogenes* infection. We therefore determined peak IFN-γ responses by NK cells upon infection. Rats were infected with *L. monocytogene*s, and bulk NK cells were analyzed for IFN-γ production by intracellular flow cytometry at the indicated times after infection. Peak responses were observed 24 hrs p.i., with a decline 48 hrs p.i. (Fig. 3A). To explore a possible influence by Ly49 receptors, we compared IFN-γ production by Ly49s3^+^ and Ly49s3^−^ NK cells. Equivalent levels of IFN-γ were produced by these subsets, suggesting that IFN-γ production is not unique to a particular subset of NK cells (Fig. 3B).

**Figure 4:**
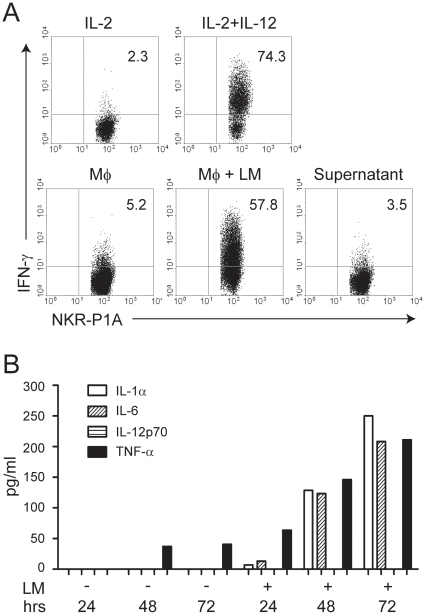
Production of IFN-γ by NK cells is cell-contact dependent. A) R2 macrophages (Mϕ) were either left untreated or infected with *L. monocytogenes* (LM) for 24 hrs prior to co-incubation with freshly isolated splenic lymphocytes for another 18 hrs. Alternatively, splenocytes were incubated with supernatants from infected R2 macrophages alone. As negative and positive controls, splenocytes were stimulated with IL-2 alone or IL-2 + IL-12, respectively. IFN-γ production by NK cells was measured by intracellular FACS staining gating on NKR-P1A^+^CD3^−^ NK cells. B) Measurement of IL-1α, IL-6, IL-12p70, and TNF-α in the supernatants of uninfected or infected R2 macrophages cultured for 24–72 hrs was done with multiplex cytokine assay analysis.

We further explored the requirements for IFN-γ production by NK cells *in vitro*. R2 macrophages were infected with *L. monocytogenes* and cultured for 24 hrs. The cells were then co-cultured with freshly isolated splenocytes for another 18 hrs in the presence of IL-2. While there was some IFN-γ production within NK cells co-cultured with uninfected macrophages, a robust IFN-γ response was observed in co-cultures with infected macrophages (Fig. 4A). To test the dependency on interaction with target cells, we cultured spleen cells with cell-free supernatants from cultures of *L. monocytogenes* infected R2 macrophages. Only low levels of IFN-γ expression was observed in NK cells, comparably to co-cultures with uninfected macrophages (Fig. 4A). Stimulation of spleen cells with IL-2 and IL-12 led to high IFN-γ production (Fig. 4A), but the amounts of IL-12 applied in this assay (2 ng/ml) may surpass the amounts produced endogenously by infected macrophages. In fact, we were unable to detect IL-12p70 in the supernatants of infected R2 macrophages, while other pro-inflammatory cytokines such as TNF-α, IL-1α, and IL-6 were induced (Fig. 4B). TNF-α is known to synergize with IL-12 in promoting IFN-γ production. Collectively, these data suggest that cell-to-cell contacts between NK cells, infected macrophages, and possibly other accessory cells are necessary for IFN-γ production.

### Down-modulation of Ly49s3 surface expression coincides with increased MHC class I expression in *L. monocytogenes* infected rats

**Figure 5:**
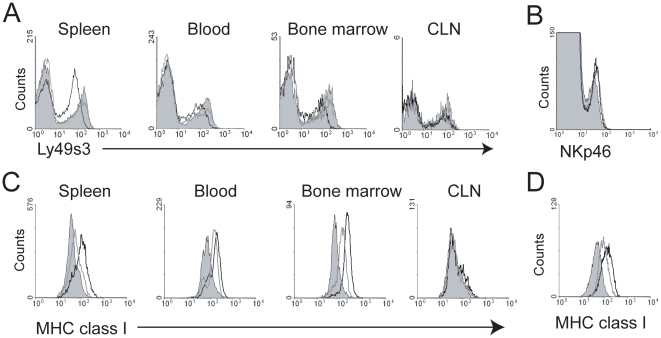
*L. monocytogenes* infection leads to up-regulation of MHC class I molecules and down-modulation of Ly49s3 surface expression on NK cells. Mononuclear leukocytes were isolated from the spleen, blood, bone marrow, and cervical lymph nodes of uninfected (filled histograms) and infected rats, Rats were infected with *L. monocytogenes* for 24 (grey line) or 48 hrs (black line). Cells were stained with antibodies and analysed for A) Ly49s3 expression on NK cells, B) NKp46^+^ expression by spleen cells, and MHC class I expression on C) OX41^+^ macrophages and D) NK cells. n = 4.

Finally, we investigated whether Ly49 receptors and their MHC class I ligands were affected by *L. monocytogenes* infection. Interestingly, we observed reduced expression levels of the Ly49s3 receptor on NK cells from spleen, blood, and bone marrow 48 hrs p.i. (Fig. 5A). This was not a general phenomenon, as Ly49s3 receptor levels remained unchanged in cervical lymph nodes (Fig. 5A). We did not observe changes in the expression of other NK cell receptors, such as NKp46 (Fig. 5B).

In rats, Ly49 receptors interact with both classical (RT1-A) and non-classical (RT1-CE) MHC class I molecules [3]–[5]. The observed down-regulation of Ly49 receptors upon *L. monocytogenes* infection prompted us to investigate the expression levels of MHC class I molecules on macrophages 24 and 48 hrs p.i. We found that expression was highly up-regulated on OX41^+^ macrophages from spleen, blood, and in the bone marrow, but not cervical lymph nodes (Fig. 5C). The same was found for splenic NK cells (Fig. 5D). Collectively, these data show that *L. monocytogenes* infection leads to increased expression of MHC class I molecules on macrophages, NK cells and possibly other cell types concomitant with a decreased surface expression of Ly49 receptors.

## Discussion

We have in a previous study provided evidence that a subset of NK cells expressing Ly49 receptors, Ly49s3^+^ NK cells, may be important for the early control of *L. monocytogenes* infection. In this study, we further explored how the Ly49s3^+^ NK cell subset is affected by *L. monocytogenes* infection.

We have previously observed that the total number of NK cells decreases in the spleen of *L. monocytogenes* infected rats, while the frequency of Ly49s3^+^ NK cells increases [29]. In this study, we found that the increased frequency of Ly49s3^+^ NK cells in the spleen was paralleled by a concomitant decrease in the bone marrow during the early phase of *L. monocytogenes* infection. This suggests that Ly49s3^+^ NK cells may migrate from the bone marrow in response to infection, and re-locate to the spleen and possibly other target organs where *L. monocytogenes* propagates such as the liver. Re-localization and migration of NK cells is likely controlled by chemokines, which are differentially expressed in tissues at steady-state and during infection [30]. During *L. monocytogenes* infection the inflammatory chemokines CCL2, CCL3, CXCL10, and CCL5 are highly expressed in the spleen and liver [31], [32], most likely produced by activated macrophages and lymphocytes [33]. In line with these data, we show here that CCL2 and CCL3, but not CXCL10 were secreted by the rat macrophage cell line R2 infected *in vitro* with *L. monocytogenes*. Interestingly, we observed that CCR5, the receptor for CCL3, and CXCR3, the receptor for CXCL10, were expressed at slightly higher levels on Ly49s3^+^ than Ly49s3^−^ NK cells in the bone marrow. This could partly explain the increased frequencies of Ly49s3^+^ NK cells found in the spleen. Both CCL3 and CXCL10 are potent chemo-attractants for NK cells [34], [35], and have been implicated in the recruitment of NK cells to the liver and lungs during infections as well as their egress from the bone marrow [36]–[38]. We also show that supernatants from *L. monocytogenes* infected R2 macrophages stimulate chemotactic responses by splenic NK cells, probably dependent on CCL3 and/or CCL2. The observed up-regulation of CXCR3 and CCR5 on NK cells in both the bone marrow and the spleen after infection, suggests that these receptors contribute to the recruitment of NK cells to the spleen during *L. monocytogenes* infection. However, the contribution of chemokine receptors may be redundant, and CCR5 is in another study suggested to be dispensable for innate immune responses to *L. monocytogenes* [39]. CCR2 is probably also involved as it has been implicated in the recruitment of monocytes during *L. monocytogenes* infection [32].

The role of Ly49 receptors in the resistance to *L. monocytogenes* has not been investigated in detail, although we have previously demonstrated that depletion of the Ly49s3^+^ NK cells increased bacterial loads in the spleen [29]. However, rat NK cells appear to produce IFN-γ independently of Ly49 receptors, as Ly49s3^+^ and Ly49s3^−^ splenic NK cells produced equivalent amounts of IFN-γ. Interestingly, we observed diminished Ly49s3 expression on NK cells 48 hrs p.i. This coincided with increased expression of MHC class I molecules on macrophages, although the up-regulation of MHC class I molecules was more rapid than the down-modulation of Ly49s3 receptors. This effect was observed in the spleen, blood, and bone marrow, but not in cervical lymph nodes. The up-regulation of MHC-I expression is intriguing, as in most infections, a down-modulation is observed. However, it is known that MHC class I molecules may be up-regulated in response to certain viruses and as well as soluble factors released during infections; such as type I and II interferons and TNF-α [14]. Down-modulation of Ly49 receptor expression has been well documented when appropriate MHC class I ligands are present [7], [9], [10], [40]. In our study, we found that MHC class I molecules are up-regulated both on macrophages and NK cells themselves, suggesting that down-modulated Ly49s3 expression may result from both *trans* and *cis* interactions with MHC class I molecules. Whether the observed down-regulation of Ly49s3 receptors affects NK cell functions, or is just a bystander effect of increased MHC class I expression is presently unclear. We can not exclude that *L. monocytogenes* might also modulate other NK cell receptors of importance in the defence against intracellular pathogens, such as NKG2/CD94 or NKG2D.

IFN-γ production by NK cells is thought to be the major protective factor against early *L. monocytogenes* infection [19]. We show here that the production of IFN-γ from naïve NK cells requires contact with infected macrophages, and that soluble factors released by *L. monocytogenes* infected macrophages are not sufficient for NK cell activation. It was recently suggested that three components are necessary for IFN-γ production by NK cells: i) Direct contact with infected cells, ii) The presence of IL-2 or IL-15 as priming factors secreted by bystander DC or T cells, and iii) IL-12 or IL-18 released from the infected or activated macrophages [23]–[25]. It is not clear why direct contact with infected cells is necessary, although directed secretion of cytokines across synapses [41], [42] or specific interactions with one or more receptors may be possible explanations [24]. Surprisingly, we did not detect any IL-12 in the supernatants of *L. monocytogenes* infected R2 macrophages. However, TNF-α may also alternatively contribute to the production of IFN-γ by NK cells [22].

In conclusion, we show that *L. monocytogenes* infected rats display an increased frequency of Ly49s3^+^ NK cells in the spleen with accompanying reductions in the bone marrow. We observed a small up-regulation of CXCR3 and CCR5 on Ly49s3^+^ NK cells that may cause a putative efflux from the bone marrow during *L. monocytogenes* infection, but this needs to be further addressed in future studies. We also observed an increased expression of MHC class I molecules on macrophages upon *L. monocytogenes* infection that may render them more sensitive to killing by CD8^+^ T cells and possibly NK-cells through their activating Ly49 receptors, although this has not yet been tested.

## Materials and Methods

### Rats

Eight to 12-week-old PVG-RT7^b^ rats [*RT1^c^* or *c*; i.e., *RT1-A^c^ -B*/*D^c^ -CE/N*/*M^c^* (class Ia-class II-class Ib), abbreviated *c-c-c*] have been bred at the Institute of Basic Medical Sciences for more than 20 generations. The Department of Comparative Medicine at the Institute of Basic Medical Sciences led by the institutional veterinarian has established the rules for feeding, monitoring handling, and sacrifice of animals in compliance with regulations set by the Ministry of Agriculture of Norway and “The European Convention for the Protection of Vertebrate Animals used for Experimental and other Scientific Purposes”. The institutional veterinarian has delegated authority from the Norwegian Animal Research Authority (NARA). The laboratory animal facilities are subject to a routine health-monitoring program and tested for infectious organisms according to a modification of Federation of European Laboratory Animal Science Associations (FELASA) recommendations. In our experiments rats were anesthetized with fentanyl citrate and fluanisone (Hypnorm, Vetapharma, UK), and sacrified by asphyxiation with CO_2_. The licence number for retrieval of organs from untreated animals is VIT09.1512. The *in vivo* experiments were approved by the Experimental Animal Board under the Ministry of Agriculture of Norway with license ID 704.

### Cells and cell cultures

Mononuclear cells from spleen and blood were prepared by Lymphoprep separation for 20 min at 600 g. Splenocytes were depleted of B cells using sheep anti-rat IgG Dynabeads® (70 µl/2×10^7^ cells; Invitrogen Dynal, Oslo, Norway). Leukocytes from cervical lymph nodes and bone marrow from femora were harvested and passed through a 70-µm cell strainer. The R2 macrophage cell line, a kind gift from Dr. Jan. G. M. C. Damoiseaux, the Netherlands, was generated by silica injection into the pleural cavity of outbred Wistar rats [43]. The cells were maintained in complete RPMI (cRPMI; RPMI 1640 supplemented with 10% foetal bovine serum, 1% penicillin/streptomycin, 1% sodium pyruvate, and 2-mercaptoethanol (all from Invitrogen, Paisley, UK)), and split every second day.

### Bacteria and infection

*Listeria monocytogenes* (strain L 242/73 type 4b, originally a gift from A. de Klerk, Department of Toxicology, Pathology and Genetics, National Institute of Public Health and the Environment (RIVM), Bilthoven, The Netherlands [44]) were grown in tryptic soy broth until mid-log phase as previously described [29]. After addition of glycerol (15% v/v), 2 ml aliquots were stored frozen at −80°C until use. Rats were injected intravenously (i.v.) in the lateral tail vein with 2×10^4^ CFU of *L. monocytogenes,* and sacrificed at 1 or 2 days post infection (p.i.). Cells were infected at a multiplicity of infection (MOI) of 5∶1 at 37°C for 1 hr, followed by washing with RPMI and resuspension in 10 µg/ml Gentamycin-containing (Sigma-Aldrich) cRPMI (without penicillin/streptomycin) to kill extracellular bacteria. The infected cells were harvested after 48–72 hrs. Giemsa stained (Sigma-Aldrich) cytospins were routinely analyzed to monitor the success of infection.

### Flow cytometry

One million cells were labelled with combinations of the following mAbs against: CD3 (G4.18-FITC or -PE), NKR-P1A (10/78-PE), CCR5 (CD195-biotin), and biotin (Streptavidin-PerCP) (all from BD Biosciences, Franklin Lakes, NJ), NKR-P1A (3.2.3-FITC; from J.C. Hiserodt, Pittsburgh, PA [45]), Ly49s3/i3/s4/i4 (DAR13-biotin, or -Alexa647 [4]), NKp46 (Wen23-biotin; a kind gift from Dr. S. Fossum, Oslo [46]), MHC class I (OX18-biotin), CD25 (OX39-biotin), and SIRPα (OX41-biotin) (kind gifts from Dr. A. Williams, MRC, Cellular Immunology Unit, Oxford). Polyclonal goat anti-CXCR3 (Y-16) and -CXCR4 (G-19), both from Santa Cruz Biotechnologies (Santa Cruz, CA), were conjugated by us to Alexa488 (Invitrogen) according to standard protocols. Samples were analyzed with a FACSCalibur (BD Biosciences). NK cells were defined and gated as NKR-P1A^+^CD3^−^, unless stated otherwise.

### Intracellular IFN-γ staining

For *in vitro* experiments, mononuclear splenocytes depleted of B cells were incubated with either uninfected or *L. monocytogenes* infected R2 macrophages in the presence of IL-2 [47] for 18 hrs. Splenocytes stimulated with IL-12 (2 ng/ml; Invitrogen) and IL-2 were used as a positive control. Brefeldin A (Sigma-Aldrich) was added at 10 µg/ml for the last 4 hrs of the stimulation, to prevent cytokine secretion. Cells were harvested and surface stained for 4-colour analysis with antibodies against NKR-P1A, CD3, and Ly49s3. Afterwards, the cells were fixed for 10 min in 2% paraformaldehyde, permeabilized with 0.5% saponin in PBS for 20 min, and then stained with PE-conjugated anti-rat IFN-γ (BD Biosciences). We tested the *in vivo* production of IFN-γ by splenic leukocytes isolated from either uninfected or *L. monocytogenes* infected rats, following the staining procedure described above. NK cells were gated as NKR-P1A^+^CD3^−^ cells.

### Chemotaxis assay

NK cells were enriched from splenocytes by Lymphoprep separation and nylon wool (G. Kisker GbR, Steinfurt, Germany) passage and subjected to chemotaxis using Corning® Transwell® permeable polycarbonate inserts with 5 µm pores in 24-well plates (Sigma-Aldrich). Cells were washed in RPMI supplemented with 2% foetal bovine serum and 25 mM Hepes and resuspended at 5×10^6^ cells/ml. Cells (100 µl) were added to the inserts in the presence of medium alone, or supernatants from either uninfected or *L. monocytogenes* infected R2 macrophages in the lower compartment. Assays were performed in duplicates at 37°C and 5% CO_2_ for 2 hrs. Migrated cells in the lower compartment were harvested, stained with antibodies, and counted by flow cytometry.

### Multiplex cytokine measurement

Cell-free supernatants from either uninfected or infected R2 macrophages were harvested and frozen at −80°C until assayed. Concentrations of released cytokines were measured using a multiplex rat cytokine immunoassay (Milliplex map rat cytokine/chemokine panel, Millipore, Billerica, MA). Duplicates of 25 µl undiluted supernatants were analysed with the Luminex xMAP platform (Bio-Rad, Hercules, CA).

### Statistical analysis

Graphics and statistical analysis were performed with the GraphPad Prism software. Data are presented as the mean ± standard error of the mean (SEM). Comparisons within an experimental group were performed with the One-way analysis of variance (ANOVA) and a *post hoc* Tukey's multiple comparisons test to compare treated to untreated samples. *P* values less than 0.05 were considered statistically significant.
